# Immunomodulatory effects of umbilical mesenchymal stem cell-derived exosomes on CD4^+^ T cells in patients with primary Sjögren's syndrome

**DOI:** 10.1007/s10787-023-01189-x

**Published:** 2023-04-03

**Authors:** Dan Ma, Zewen Wu, Xingxing Zhao, Xueqing Zhu, Qi An, Yajing Wang, Jingwen Zhao, Yazhen Su, Baoqi Yang, Ke Xu, Liyun Zhang

**Affiliations:** 1 Shanxi Bethune Hospital, Shanxi Academy of Medical Sciences, Third Hospital of Shanxi Medical University, Taiyuan, 030032 Shanxi China; 2 Shanxi University of Chinese Medicine, Jinzhong, 030619 Shanxi China

**Keywords:** Mesenchymal stem cell, Exosome, Sjögren's syndrome, CD4^+^ T cell, Autophagy

## Abstract

**Background:**

Primary Sjögren's syndrome (pSS) is an autoimmune disease that leads to the destruction of exocrine glands and multisystem lesions. Abnormal proliferation, apoptosis, and differentiation of CD4^+^ T cells are key factors in the pathogenesis of pSS. Autophagy is one of the important mechanisms to maintain immune homeostasis and function of CD4^+^ T cells. Human umbilical cord mesenchymal stem cell-derived exosomes (UCMSC-Exos) may simulate the immunoregulation of MSCs while avoiding the risks of MSCs treatment. However, whether UCMSC-Exos can regulate the functions of CD4^+^ T cells in pSS, and whether the effects via the autophagy pathway remains unclear.

**Methods:**

The study analyzed retrospectively the peripheral blood lymphocyte subsets in pSS patients, and explored the relationship between lymphocyte subsets and disease activity. Next, peripheral blood CD4^+^ T cells were sorted using immunomagnetic beads. The proliferation, apoptosis, differentiation, and inflammatory factors of CD4^+^ T cells were determined using flow cytometry. Autophagosomes of CD4^+^ T cells were detected using transmission electron microscopy, autophagy-related proteins and genes were detected using western blotting or RT-qPCR.

**Results:**

The study demonstrated that the peripheral blood CD4^+^ T cells decreased in pSS patients, and negatively correlated with disease activity. UCMSC-Exos inhibited excessive proliferation and apoptosis of CD4^+^ T cells in pSS patients, blocked them in the G0/G1 phase, inhibited them from entering the S phase, reduced the Th17 cell ratio, elevated the Treg ratio, inhibited IFN-γ, TNF-α, IL-6, IL-17A, and IL-17F secretion, and promoted IL-10 and TGF-β secretion. UCMSC-Exos reduced the elevated autophagy levels in the peripheral blood CD4^+^ T cells of patients with pSS. Furthermore, UCMSC-Exos regulated CD4^+^ T cell proliferation and early apoptosis, inhibited Th17 cell differentiation, promoted Treg cell differentiation, and restored the Th17/Treg balance in pSS patients through the autophagy pathway.

**Conclusions:**

The study indicated that UCMSC-Exos exerts an immunomodulatory effect on the CD4^+^ T cells, and maybe as a new treatment for pSS.

**Supplementary Information:**

The online version contains supplementary material available at 10.1007/s10787-023-01189-x.

## Introduction

Primary Sjögren's syndrome (pSS) is an autoimmune disease that leads to the destruction of exocrine glands and multi-system lesions. Its main feature is the presence of a large number of lymphocytes, mainly CD4^+^ T cells, in exocrine glands. Abnormal proliferation, apoptosis, and differentiation of CD4^+^ T cells are key factors causing pSS immune disorders (Ramos-Casals et al. 2020; An et al. 2022). To date, the treatment of pSS includes symptomatic treatment, glucocorticoids, disease-modifying antirheumatic drugs (DMARDs), and biological agents (Ramos-Casals et al. 2020). These treatments only target active inflammation and fail to repair late damaged tissues. Therefore, there is an urgent need to develop new therapeutic strategies that can both regulate the immune disorder and repair damaged tissues.

Mesenchymal stem cells (MSCs) exhibit immunomodulatory and tissue repair abilities (Chansaenroj et al. 2021). Extracellular vesicles (EVs), including exosomes (Exos), microparticles, and apoptotic bodies, are released by MSCs (Zhao et al. 2022). Exos can simulate the effects of MSCs, and the immunomodulatory effect is stronger than that of microparticles (Cosenza et al. 2018). Compared with MSCs, Exos can quickly pass through capillaries, have stable properties, and a strong information transmission ability. Furthermore, ectopic osteogenesis (Fennema et al. 2018), tumor formation (Jeong et al. 2011), pulmonary capillary interception (Wang et al. 2015), and immune rejection (Lohan et al. 2017) caused by cell therapy can be avoided by using Exos, which is expected to become a new strategy. MSC-EVs derived from human-induced pluripotent stem cells (Hai et al. 2018), mouse olfactory MSC-Exos (Rui et al. 2021), and human labial gland MSC-Exos (Li et al. 2021) can increase the salivary flow rate in the pSS animal model of non-obese diabetic (NOD) mice, reduce salivary gland lymphocyte infiltration, inhibit Th17 cell differentiation, and induce regulatory T cell (Treg) differentiation. Compared to other sources, umbilical cord-derived MSCs (UCMSCs) have low immunogenicity (Barcia et al. 2015), strong proliferation (Trivanovic et al. 2015), great differentiation (Trivanovic et al. 2015), and a strong immunomodulatory ability (Kim et al. 2018). They are the most promising stem cells in clinical applications. Studies have demonstrated that UCMSCs can reduce salivary gland lymphocyte infiltration in NOD mice and inhibit the proliferation of T cells in pSS patients (Liu et al. 2021; Alunno et al. 2015). However, whether UCMSC-Exos can regulate the function of CD4^+^ T cells and specific mechanisms remains unclear.

Autophagy refers to the degradation of damaged organelles and macromolecules by lysosomes under the regulation of autophagy-related genes (ATGs). Autophagy is a key mechanism regulating the proliferation, apoptosis, and differentiation of CD4^+^ T cells, and maintains immune homeostasis and function (Clarke and Simon 2019). However, studies on autophagy in pSS have mainly focused on glandular epithelial cells, while little is known about its function in CD4^+^ T cells (Katsiougiannis et al. 2015; Voynova et al. 2020; Alessandri et al. 2017). Additionally, previous studies on MSCs in pSS have mainly focused on their effects on T cell functions that are mediated by regulating the cytokine microenvironment (Matsumura-Kawashima et al. 2021; Yao et al. 2019; Liu et al. 2015) or cells such as natural killer (NK) (Shi et al. 2018) and myeloid inhibitory cells (Rui et al. 2021; Tian et al. 2020). Whether MSC-Exos regulates CD4^+^ T cell functions via the autophagy pathway remains unknown.

Herein, we analyzed the peripheral blood lymphocyte subsets in pSS patients, clarified whether there were CD4^+^ T cell abnormalities, and explored the relationship between lymphocyte subsets and the European League Against Rheumatism (EULAR) SS disease activity index (ESSDAI). Next, we investigated whether UCMSC-Exos could regulate the proliferation, apoptosis, differentiation, and inflammatory factor secretion of CD4^+^ T cells, and that via autophagy. This study provides an important basis for exploring UCMSC-Exos as a new treatment for pSS.

## Materials and methods

### Population

The clinical data of 60 pSS patients and 22 age- and gender-matched non-Sjögren syndrome (nSS) patients with dry eyes or mouth symptoms were collected from the Department of Rheumatology, Shanxi Bethune Hospital, from January 2021 to March 2022 retrospectively. The peripheral blood lymphocyte subsets were compared between the two groups, and the correlation between lymphocyte subsets and ESSDAI score (low, 0–4; moderate, 5–13; high, ≥ 14) (Ramos-Casals et al. 2020) was analyzed. Subsequently, 72 pSS patients, and 24 age- and gender-matched healthy subjects were selected prospectively (Supplementary table S1). 20 mL blood was obtained and sort CD4^+^ T cells. pSS patients who met the SS classification criteria established by the American College of Rheumatology (ACR)/EULAR in 2016 (Ramos-Casals et al. 2020), and had not received glucocorticoids, DMARDs, etc. during the previous six months were included. SS patients secondary to other connective tissue diseases, with acute and chronic diseases were excluded. Umbilical cords of normal full-term cesarean section neonates in the Obstetrics and Gynecology Department of Shanxi Bethune Hospital were obtained. The study was approved by the Ethics Committee of Shanxi Bethune Hospital (approval number: SBQLL-2020–030).

### Isolation and identification of UCMSCs and UCMSC-Exos

UCMSCs isolation, morphological examination, surface antigen phenotypes (FITC-CD105, PE-CD73, PC7-CD90, PC5-CD45, PC5-CD34, and PE-HLA-DR; BioLegend, San Diego, CA, USA), and the adipogenic and osteogenic abilities, were investigated as previously described (Ma et al. 2019).

DMEM/F12 medium without FBS (Gibco, Carlsbad, CA, USA) was added to the UCMSCs and cells were cultured for 24 h. The supernatant was collected and centrifuged at 300 g for 10 min, 2,000 g for 10 min, and 10,000 g for 30 min. Next, it was filtered and sterilized through a 0.22 μm filter, centrifuged at 100,000 g for 70 min, repeated once, and resuspended in 100 μL of PBS. Exos were stained with 0.2% phosphotungstate acid (Servicebio Technology Co., Ltd., Wuhan, China) and the morphology was observed using transmission electron microscopy (TEM; HITACHI Ltd., Tokyo, Japan). Particle size was determined using nanoparticle tracking analysis (NTA: Particle Metrix GmbH, Ammersee, Germany), and concentration was determined using a BCA protein quantification kit (Beyotime, Shanghai, China). Western blot was used to detect markers as follows: the SDS-PAGE gel (Solarbio Science & Technology Co., Ltd., Beijing, China) was prepared (Supplementary table S2), the sample volume was calculated on the basis of protein concentration, and each group of samples and markers were subjected to electrophoresis. The gel was cut and proteins were transferred to a polyvinylidene fluoride (PVDF) membrane. The membrane was blocked with 5% non-fat milk powder for 2 h. Then, the PVDF membrane was placed into the primary antibodies, diluted according to the manufacturer's instructions (rabbit anti-human CD9, CD81, and TSG101) (Abcam, Cambridge, UK), and incubated at 4 °C for 16 h. Then, the PVDF membrane was placed into the diluted goat anti-rabbit IgG antibody (Abcam) solution, incubated for 1 h at 4 °C, and washed with TBST. The color was developed using the horseradish peroxidase-enhanced chemiluminescence method. The gray value of the protein band was analyzed using the Image J analysis (V1.8.0) and the relative protein expression level of the protein samples was calculated according to the ratio of the gray value to the internal reference.

### Immunomagnetic separation of CD4^+^ T cells

Peripheral blood mononuclear cells (PBMCs) were separated, resuspended in 80 μL magnetic bead sorting buffer, and added to 20 μL CD4^+^ T cell magnetic beads (Miltenyi Biotec, Miltenyi Biotec, Bergisch Gladbach, Germany). The cells were centrifuged at 1,600 rpm for 10 min, resuspended in 500 µL buffer, and added to the sorting column. The flow-through fraction contained the CD4^−^ T cells, while the CD4^+^ T cells remained in the tube.

A 24-well plate was coated with anti-CD3 antibody (5 μg/mL; BioLegend), CD4^+^ T cells were added, and the number was adjusted to 5 × 10^5^ cells. PBS-resuspended UCMSC-Exos, rapamycin (RAPA; Sigma, St. Louis, MO, USA), and hydroxychloroquine (HCQ; Sigma), was added, respectively, at a total volume of 500 μL. They were divided into the healthy control (HC; PBS intervention), pSS (PBS intervention), UCMSC-Exos (90 μg/mL), RAPA (1 μmol/L), RAPA + UCMSC-Exos (1 μmol/L RAPA + 90 μg/mL UCMSC-Exos), HCQ (25 μmol/L), and HCQ + UCMSC-Exos (25 μmol/L HCQ + 90 μg/mL UCMSC-Exos) groups. One sample consisting of 2–3 pSS patient CD4^+^ T cells was divided into different intervention groups. The concentrations of UCMSC-Exos were selected according to the cell counting kit-8 (CCK8) experiment (Supplementary 1.2). The RAPA and HCQ concentrations were selected according to literature (Chen et al. 2016; van Loosdregt et al. 2013). The anti-CD28 antibody (2 μg/mL; BioLegend) was added and incubated for 72 h, and the cells and supernatant were collected.

### Flow cytometry

The lymphocyte subsets were detected using Cyto-STAT tetraCHROME CD45-FITC/CD56-RD1/CD19-ECD/CD3-PC5 (6,607,073, Beckman Coulter, Inc., Hialeah, FL, USA). For the purity of CD4^+^ T cells, we added FITC-CD4 antibody (BioLegend) in one tube and FITC-IgG1 isotype control antibody (BioLegend) in another tube. For CD4^+^ T cell cycle analysis, the cell suspension was centrifuged at 1,600 rpm for 10 min. 500 μL of 75% ethanol, which was pre-cooled at -20 °C, was added, and cells were fixed at 4 °C for 4 h, followed by centrifugation. Cells were incubated with 100 μL of RNase A solution (Solarbio Science & Technology Co., Ltd.), followed by incubation with 400 μL of propidium iodide staining solution (Solarbio Science & Technology Co., Ltd.). Flow cytometry (Beckman Coulter, Inc.) was used to detect the red fluorescence at an excitation wavelength of 488 nm. The proliferation index (PI) was calculated as follows: (S + G2/M)/(G0/G1 + S + G2/M). For CD4^+^ T cell apoptosis analysis, 1 mL binding buffer was added to the cell suspension and centrifuged. Cells were incubated with 5 μL of Annexin V-FITC and propidium iodide (PI; Solarbio).

For Th1, Th2, and Th17 analysis, 2 μL of phorbol ester/ionomycin and brefeldin A/monensin mixture (LiankeBio, Hangzhou, China) was added to cells and cocultured for 5 h. Cells were incubated with 500 μL of Foxp3 membrane breaking solution, and mouse anti-human antibodies APC-IFN-γ, PE-IL-4, and Alexa Fluor 488-IL-17A (3 μL each; BioLegend). For Treg analysis, CD4^+^ T cells were incubated with 3 μL of mouse anti-human antibody APC-CD25 (BioLegend). Then, cells were incubated with Foxp3 membrane breaking solution, and incubated with 3 μL of mouse anti-human antibody PE-Foxp3 (BioLegend). CD4^+^ T cell-related inflammatory factors in the supernatant were detected using AimPlex^®^ Bead-Based Multiplex Immunoassays (QuantoBio, Beijing, China).

### Detection of autophagy levels

The CD4^+^ T cells were stained with 2% uranium acetate and lead citrate, and autophagosomes were detected using TEM. The autophagy-related proteins and genes were detected using western blotting or RT-qPCR. The primary antibodies were rabbit anti-human BECLIN1, LC3, and β-actin antibodies (Abcam). The secondary antibody was a goat anti-rabbit IgG antibody (Abcam). The SDS-PAGE gel is described in Supplementary table S3. RNA was extracted using an EZBioscience (Roseville, MN, USA) EZ-press RNA Purification Kit, and RNA reverse transcription and cDNA amplification were performed using PrimeScript™ RT Master Mix and TB Green® Premix Ex Taq™ II kits from TaKaRa Bio Inc. (Shiga, Japan). The primers for *BECLIN 1, LC3II*, and *β-actin* were synthesized by TaKaRa Bio Inc. company (Supplementary table S4). A Bio-Rad C1000 PCR instrument (Bio-Rad Laboratories, Inc., Hercules, CA, USA) was used for detection. The relative expressions of *Beclin1* and *LC3II* were calculated using the 2-^△△ CT^ method.

### Statistical analysis

SPSS 25.0 was used for analysis. Data are expressed as the mean ± standard deviation, the median (quartile), or rate. Comparison among different groups were performed using analysis of variance, or Kruskal–Wallis tests. Correlation analysis was performed using either the Pearson or Spearman methods. *P* values of < 0.05 were considered statistically significant.

## Results

### Lymphocyte subsets in pSS patients

**Fig. 1:**
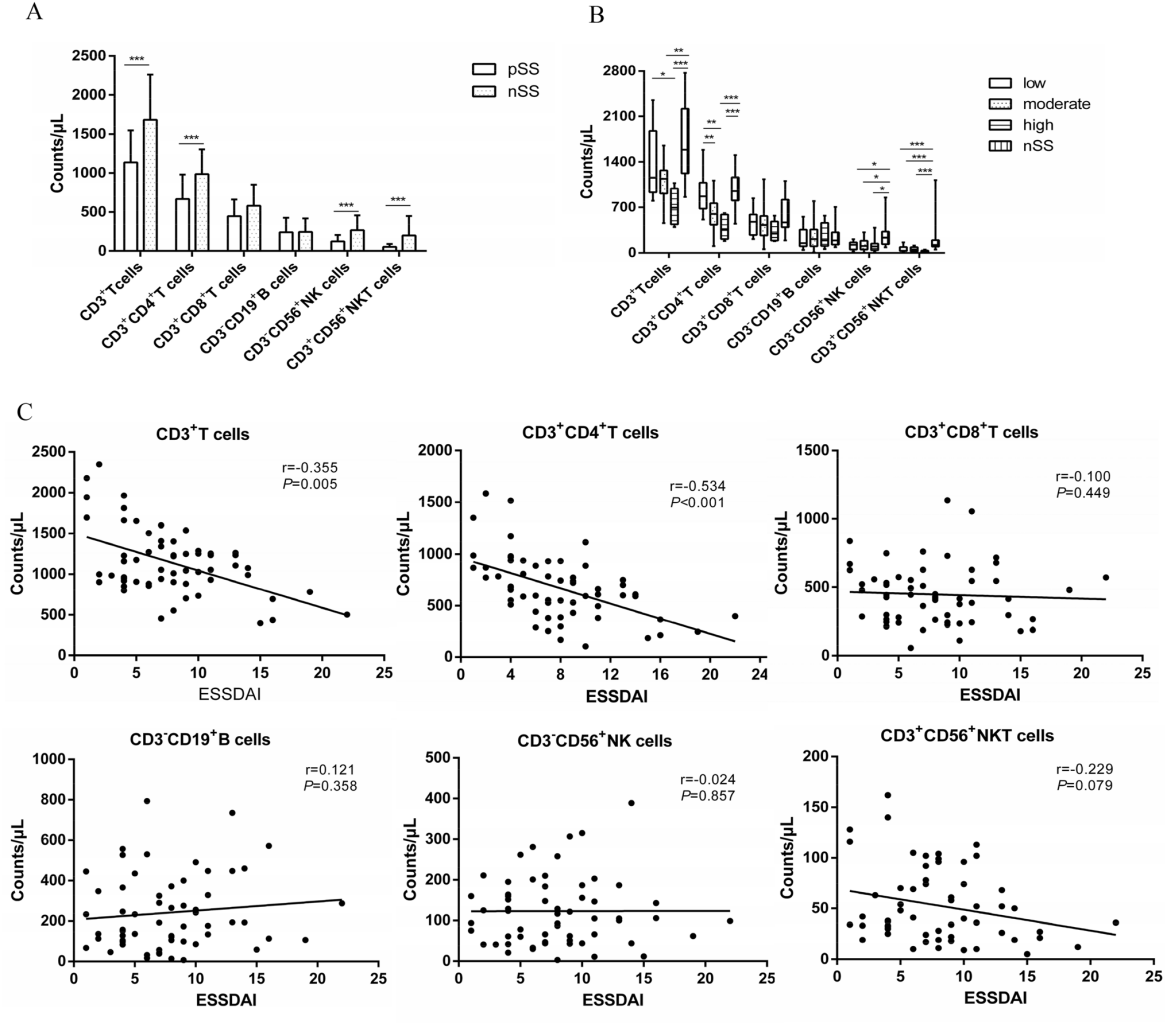
The peripheral blood lymphocyte subsets in pSS patients. **A** Comparison of lymphocyte subsets between pSS patients and nSS patients. **B** Comparison of lymphocyte subsets between pSS patients with different ESSDAI score and nSS patients. **C** The correlation between lymphocyte subsets and ESSDAI score in pSS patients. *P < 0.05, **P < 0.01, ***P < 0.001

The characteristics of pSS patients are shown in Table 1. There were 20 females, and 2 males in the nSS group, with an age range of 54.27 ± 9.33 years. Compared to nSS group, the CD3^+^ T-, CD3^+^CD4^+^ T-, CD3^−^CD56^+^ NK-, and CD3^+^CD56^+^ Natural Killer T (NKT) cells were decreased (Fig. 1A); CD3^−^CD56^+^ NK- and CD3^+^CD56^+^ NKT cells were decreased in the low disease activity group, and CD3^+^ T-, CD3^+^CD4^+^ T-, CD3^−^CD56^+^ NK-, and CD3^+^CD56^+^ NKT cells were decreased in the moderate and high disease activity group (Fig. 1B). The CD3^+^CD8^+^ T- and CD3^−^CD19^+^ B cells were not significantly different (Fig. 1A,B). The correlation analysis showed a negative correlation between CD3^+^ T cells, CD3^+^CD4^+^ T cells and ESSDAI. Other subsets showed no correlation with ESSDAI (Fig. 1C).

**Table 1:** Characteristics of the patients with pSS

Characteristics				Laboratory examination	
Age, mean ± SD, years	50.58 ±11.30	Cutaneous vasculitis, n (%)	4 (6.7%)	UWSF* ≤0.1 ml/min, n (%)	36 (60.0%)
Gender (female/male)	57/3	ILD*, n (%)	18 (30.0%)	Schirmer test ≤5 mm/5min, n (%)	30 (50.0%)
Duration, M (Q25, Q75)*, years	2.50 (1.00, 5.75)	RTA*, n (%)	8 (13.3%)	Ocular stain (+), n (%)	37 (61.7%)
Ocular/oral symptoms, n (%)	39 (65.0%)	PNS*, n (%)	2 (3.3%)	High IgG, n (%)	39 (65.0%)
Fever, n (%)	3 (5.0%)	Hematological system	34 (56.7%)	RF* (+) , n (%)	25 (41.7%)
Lymphadenopathy, n (%)	13 (21.7%)	Leucocytopenia , n (%)	16 (26.7%)	Anti-SSA/Ro52 antibody (+), n (%)	47 (78.3%)
Glandular swelling, n (%)	3 (5.0%)	Anaemia, n (%)	15 (25.0%)	Anti-SSA/Ro60 antibody (+), n (%)	46 (76.7%)
Joint swelling/pain, n (%)	10 (16.7%)	Thrombocytopenia, n (%)	7 (11.7%)	Anti-SSB antibody (+), n (%)	36 (60.0%)
				Biopsy focus (+), n (%)	40 (66.7%)
ESSDAI*					
ESSDAI (1–4), n (%)		17 (28.3%)			
ESSDAI (5–13), n (%)		36 (60.0%)			
ESSDAI (≥ 14), n (%)		7 (11.7%)			

*ESSDAI European League Against Rheumatism Sjögren’s syndrome disease activity index, *ILD* interstitial lung disease, *M* median, *pSS* primary Sjogren's Syndrome, *PVS* peripheral nervous system, *Q* quartile, *RF* rheumatoid factor, *RTA* renal tubular acidosis, *SD* standard deviation, *UWSF* unstimulated whole salivary flows

### Identification of UCMSCs and UCMSC-Exos

**Fig. 2:**
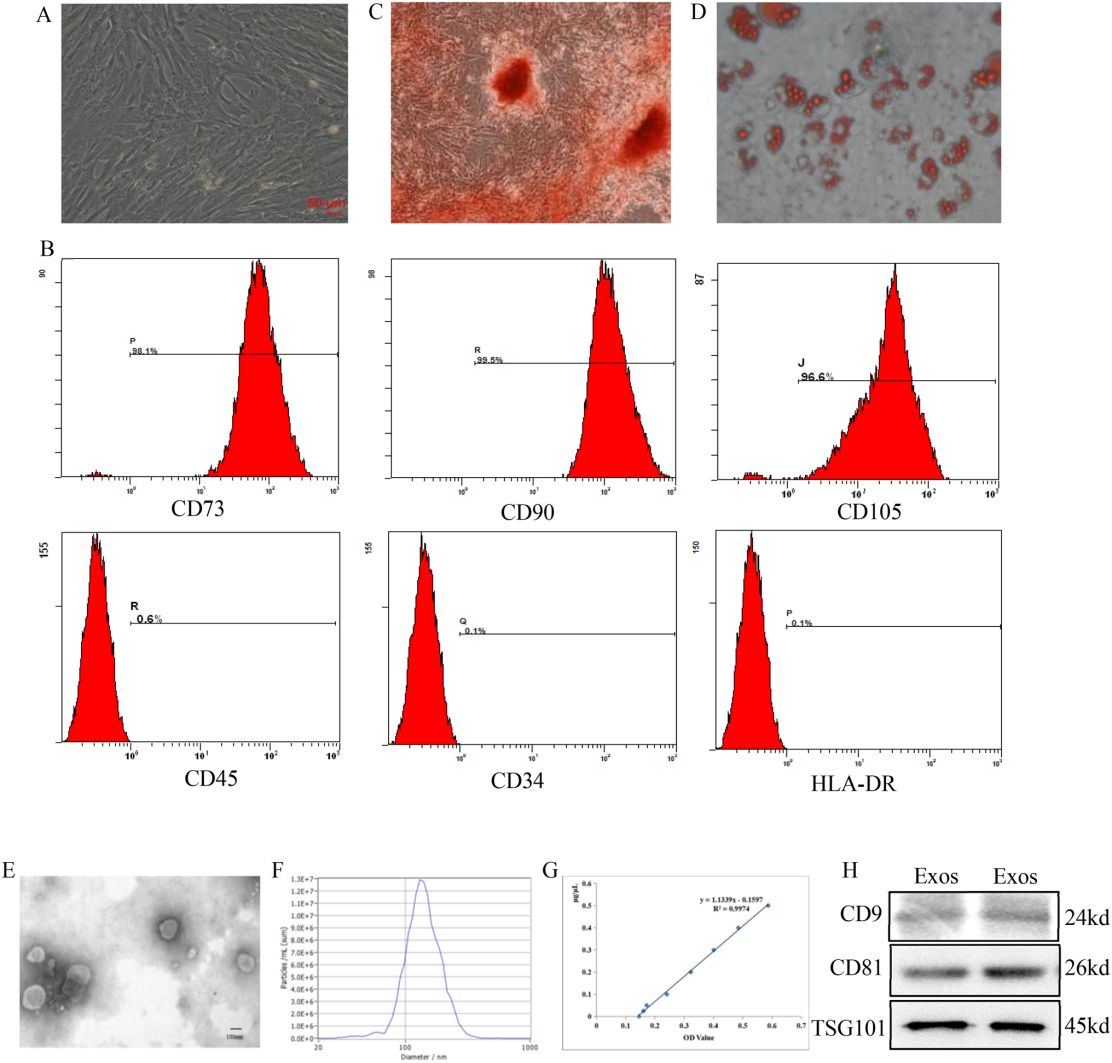
The identification of UCMSCs and UCMSC-Exos. **A** UCMSCs are fibrous in shape (× 100). **B** UCMSCs highly express CD73, CD90, and CD105, with a cell ratio of > 90%, but show low expression of CD45, CD34, and HLA-DR, with a cell ratio of ≤ 2%. **C** UCMSCs are able to differentiate into osteoblasts indicated by alizarin red staining (× 100). **D.** UCMSCs are able to differentiate into adipocytes indicated by Oil red O staining (× 100). **E** TEM showed that UCMSC-Exos were vesicle-like structure surrounded by a lipid bimolecular structure (× 50 000). **F** NTA determined the particle diameter (144.4 ± 44.0 nm), median diameter (137.7 nm), and concentration (2.0 × 10^11^ particles/mL). **G** The protein concentration determined using the BCA method was 1.00 μg/μL. **H** Western blot showed that the UCMSC-Exos expressed CD9, CD81, and TSG101

UCMSCs are plastic-adherent and fibrous in shape (Fig. 2A). The cells highly expressed CD73, CD90, and CD105, but showed low expression of CD45, CD34, and HLA-DR (Fig. 2B). These cells were able to differentiate into osteoblasts and adipocytes in vitro (Fig. 2C,D). UCMSC-Exos were vesicle-like structures surrounded by a lipid bimolecular structure (Fig. 2E). NTA determined the particle diameter (144.4 ± 44.0 nm), median diameter (137.7 nm), and concentration (2.0 × 10^11^ particles/mL) (Fig. 2F). The protein concentration was 1.00 μg/μL (Fig. 2G). UCMSC-Exos expressed CD9, CD81, and TSG101 (Fig. 2H).

### Effects of UCMSC-Exos on CD4^+^ T cells

The purity of CD4^+^ T cells sorted by immunomagnetic beads was 98.29% (Supplementary Fig S1). The CCK8 result found that 90 μg/mL UCMSC-Exos significantly inhibited the peripheral blood CD4^+^ T cell proliferation of pSS patients, better than 30 or 60 μg/mL. Therefore, the subsequent experiments were intervened with 90 μg/mL (Supplementary Fig S2).

**Fig. 3:**
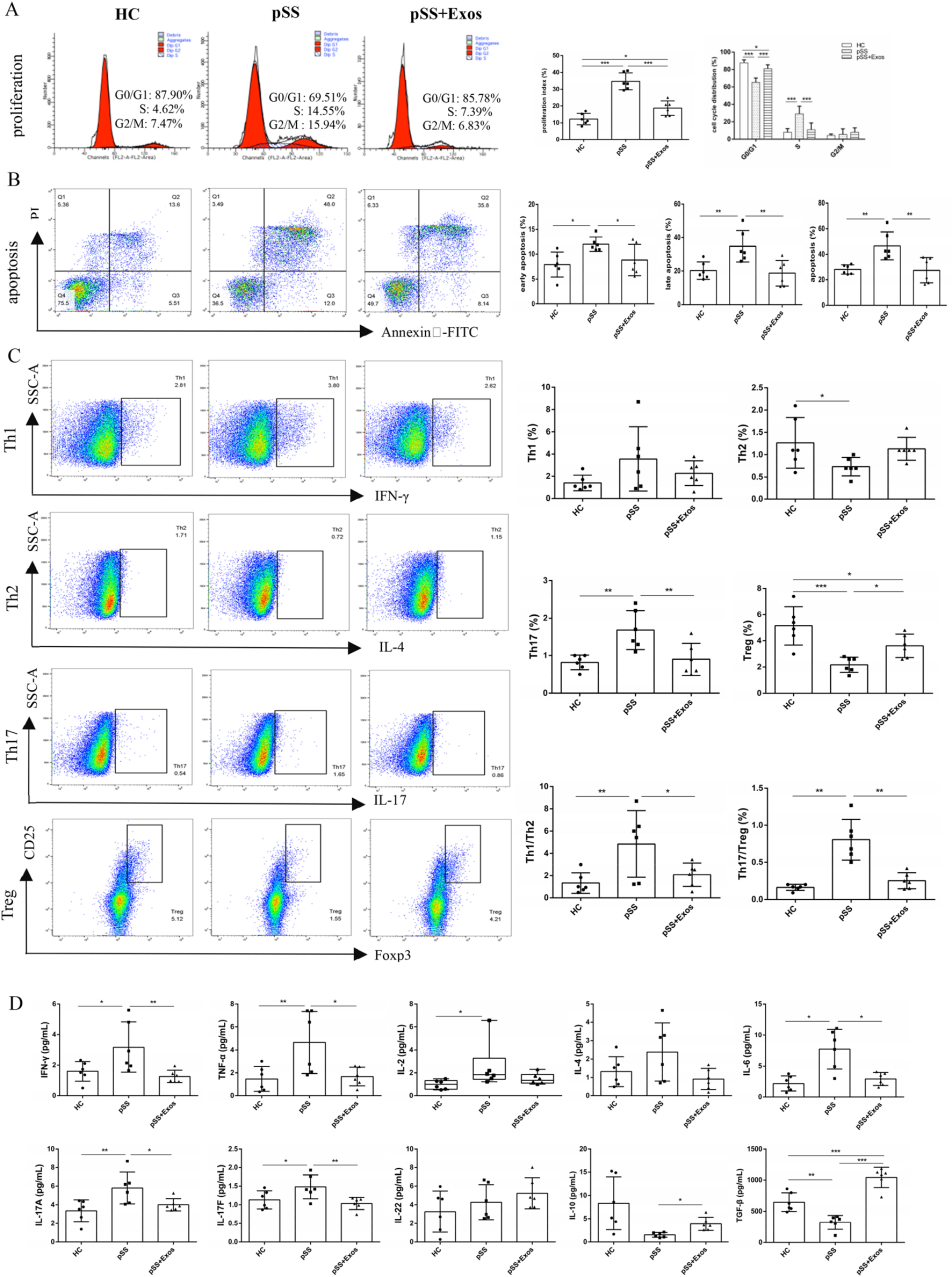
Effects of UCMSC-Exos on CD4^+^ T cells proliferation, apoptosis, differentiation, and inflammatory factors in the peripheral blood of pSS patients. **A.** UCMSC-Exos inhibited the hyperproliferation of CD4^+^ T cells, blocked them in the G0/G1 phase, and inhibited them from entering the S phase. **B** UCMSC-Exos inhibited the excessive apoptosis of CD4^+^ T cells. **C** UCMSC-Exos inhibited the differentiation of CD4^+^ T cells to Th17 cells and Tregs, and restored the balance of Th1/Th2 and Th17/Tregs. **D** UCMSC-Exos inhibited the secretion of the CD4^+^ T cell-related pro-inflammatory factors IFN-γ, TNF-α, IL-6, IL-17A, and IL-17F, and promoted the secretion of anti-inflammatory factors IL-10 and TGF-β. *P < 0.05, **P < 0.01, ***P < 0.001

Compared to the HC group, the proliferation of CD4^+^ T cells was higher in the pSS group and decreased after UCMSC-Exos intervention (Fig. 3A); the proportion of CD4^+^ T cells in the G0/G1 phase was reduced while that in the S phase was increased. Compared to the pSS group, the cells in the G0/G1 phase of the UCMSC-Exos group was increased, while the cells in the S phase was decreased. The cells in the G0/G1 phase of the UCMSC-Exos group was slightly lower than that in the HC group. The proportion showed no significant difference in the G2/M phase (Fig. 3A). This indicated that UCMSC-Exos inhibited CD4^+^ T cell hyperproliferation in pSS patients, blocked them in the G0/G1 phase, and inhibited them from entering the S phase.

To avoid the effect of necrotic cells on apoptosis, the early, late, and overall apoptosis of CD4^+^ T cells was calculated. The apoptosis of CD4^+^ T cells in the pSS group was higher than that in the HC group, whereas that in the UCMSC-Exos group was lower than that in the pSS group and not statistically different from that in the HC group (Fig. 3B). This indicated that UCMSC-Exos inhibited the excessive apoptosis of CD4^+^ T cells in pSS patients.

No significant differences in the Th1 cell ratios among the HC, pSS, and UCMSC-Exos groups. Compared with the HC group, the proportion of Th2 cells and Tregs in the pSS group decreased, while Th17 cells, Th1/Th2, and Th17/Treg ratios increased. Compared with the pSS group, the proportion of Th2 cells in the UCMSC-Exos group tended to increase, but not with statistical significance; the proportion of Tregs was increased, while Th17 cells, Th1/Th2, and Th17/Treg ratios decreased. There were no differences in the Th2, Th17, Th1/Th2, and Th17/Treg ratios between the HC and UCMSC-Exos groups; however, the proportion of Tregs in the UCMSC-Exos group was lower than that in the HC group (Fig. 3C). These results suggest that abnormal CD4^+^ T cell differentiation in pSS patients causes Th1/Th2 and Th17/Treg imbalances, whereas UCMSC-Exos restored Th1/Th2 and Th17/Treg balances.

Compared with the HC group, the expressions of IFN-γ, TNF-α, IL-2, IL-6, IL-17A, and IL-17F in the pSS group were increased, TGF-β was decreased, and IL-10 showed a declining trend. Compared with the pSS group, the expression of IFN-γ, TNF-α, IL-6, IL-17A, and IL-17F in the UCMSC-Exos group decreased, IL-10 and TGF-β increased, and IL-2 showed a non-significant downward trend. The TGF-β expression in the UCMSC-Exos group was higher than that in the HC group; IL-4 and IL-22 levels were not significantly different (Fig. 3D). This indicated that UCMSC-Exos regulated the secretion of inflammatory factors in pSS patients.

### Effects of UCMSC-Exos on CD4^+^ T cells autophagy

**Fig. 4:**
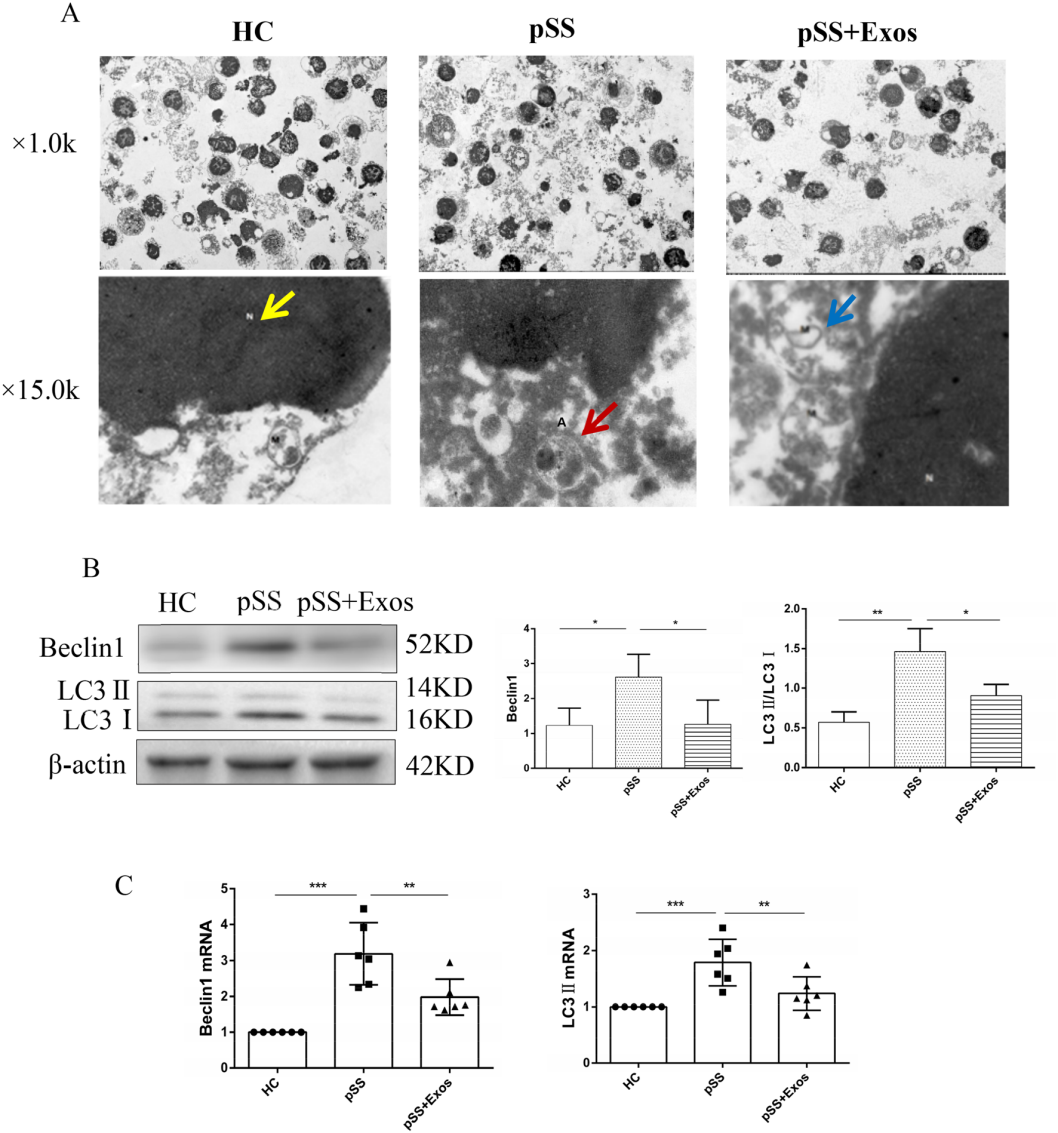
Effects of UCMSC-Exos on CD4^+^ T cells autophagy levels. **A** The autophagolysosome structure, which was surrounded by a monolayer membrane and contained organelles or cytoplasmic components, was observed in the pSS group using TEM, but was not observed in the HC or UCMSC-Exos groups. (N: cell nucleus, shown by yellow arrow; M: mitochondria, shown by blue arrow; A: autophagosome, shown by red arrow; × 15.0 k). **B**, **C** UCMSC-Exos decreased the levels of Beclin1 and LC3II/LC3I (**B**), *Beclin1* mRNA and *LC3II* mRNA (**C**) on CD4^+^ T cells in the peripheral blood of pSS patients *P < 0.05, **P < 0.01, ***P < 0.001

The autophagosome structure, which was surrounded by a doublelayer membrane and contained organelles or cytoplasmic components, was observed in the pSS group, but not in the HC or UCMSC-Exos groups (Fig. 4A). BECLIN1, LC3II/LC3I, *BECLIN1* mRNA, and *LC3II* mRNA levels were increased in the pSS group compared with those in the HC group. UCMSC-Exos decreased those levels in pSS patients but those of the HC group showed no differencev(Fig. 4B, C). This suggested that CD4^+^ T cell autophagy in pSS patients was increased and that it could be reduced by UCMSC-Exos.

### UCMSC-Exos exert immunoregulatory effects on CD4^+^ T cells through the autophagy pathway

To demonstrate that the concentration of RAPA and HCQ can cause changes in autophagy levels, we firstly detected the autophagy proteins; the result showed that BECLIN1 and LC3II/LC3I expression increased after RAPA intervention, and decreased after HCQ intervention (Supplementary Fig S3).

**Fig. 5:**
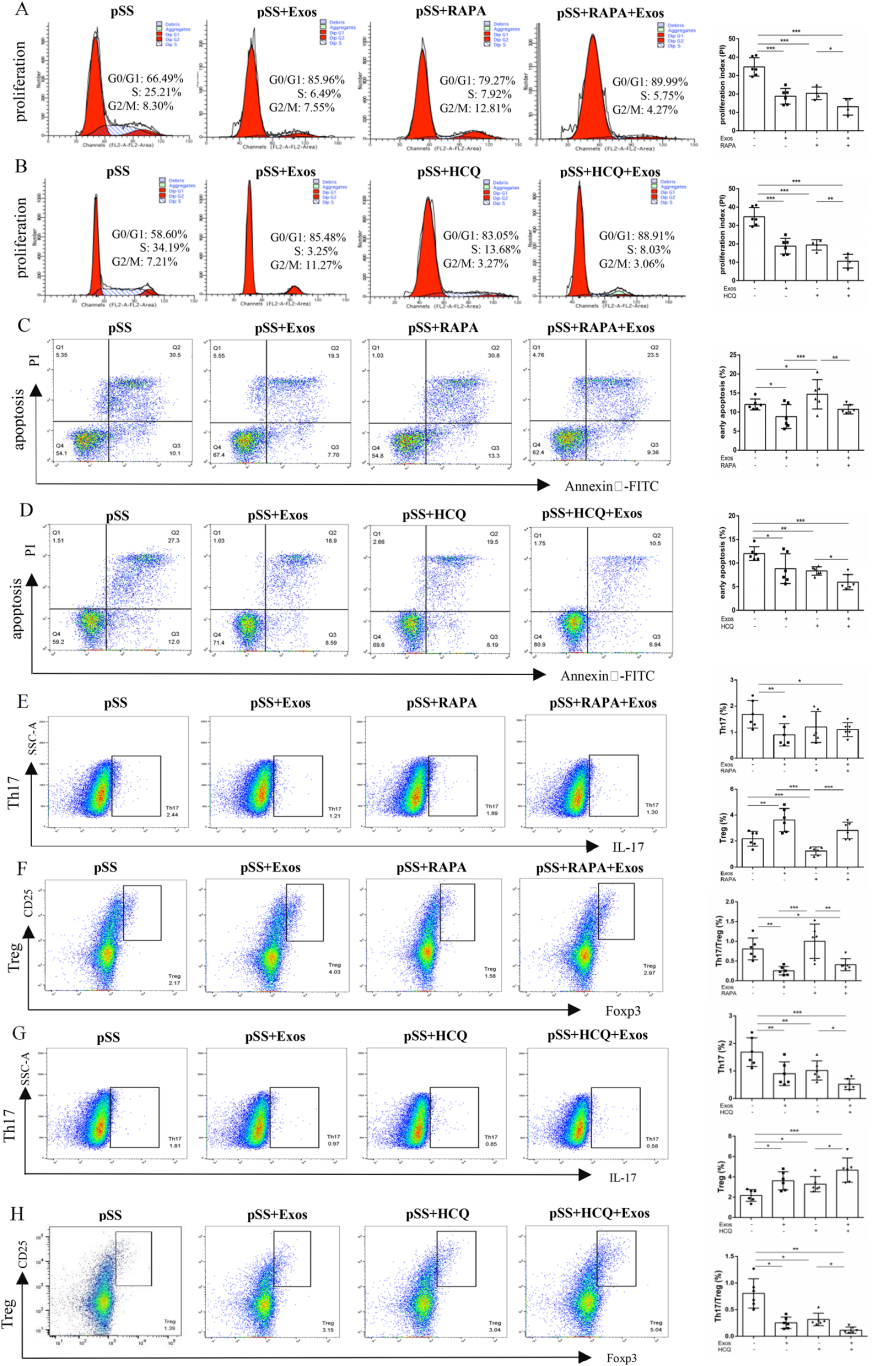
UCMSC-Exos regulated the proliferation, apoptosis, and differentiation of CD4^+^ T cells in pSS patients by inhibiting the autophagy. **A** Effects of UCMSC-Exos on CD4^+^ T cells proliferation after autophagy induction by RAPA. **B** Effects of UCMSC-Exos on CD4^+^ T cells proliferation after autophagy inhibited by HCQ. **C** Effects of UCMSC-Exos on CD4^+^ T cells apoptosis after autophagy induction by RAPA. **D** Effects of UCMSC-Exos on CD4^+^ T cells apoptosis after autophagy inhibited by HCQ. **E**, **F** Effects of UCMSC-Exos on Th17 cells (**E**) and Tregs differentiation (**F**) after RAPA induction of autophagy. **G**, **H** Effects of UCMSC-Exos on Th17 cells (**G**) and Tregs differentiation (**H**) after HCQ inhibition of autophagy. *P < 0.05, **P < 0.01, ***P < 0.001

Compared to the pSS group, the proliferation of CD4^+^ T cells in the UCMSC-Exos, RAPA, and RAPA + UCMSC-Exos groups decreased, while that in the UCMSC-Exos and RAPA groups did not differ. Compared to the RAPA group, the proliferation of CD4^+^ T cells in the RAPA + UCMSC-Exos group was further decreased (Fig. 5A). Upon autophagy induction CD4^+^ T cells with RAPA, the proliferation of CD4^+^ T cells decreased, contrary to the expected results of enhanced proliferation with increased autophagy. Therefore, CD4^+^ T cells were further treated with the autophagy inhibitor HCQ. Compared to the pSS group, the proliferation of CD4^+^ T cells in the UCMSC-Exos, HCQ, and HCQ + UCMSC-Exos groups decreased. No difference was observed between the UCMSC-Exos and HCQ groups. Compared to the HCQ group, the proliferation of CD4^+^ T cells in the HCQ + UCMSC-Exos group was further decreased (Fig. 5B). The results suggested that UCMSC-Exos inhibited CD4^+^ T cell hyperproliferation by inhibiting autophagy.

Compared with the pSS group, the early apoptosis of CD4^+^ T cells was decreased in the UCMSC-Exos group, increased in the RAPA group, and unaltered in the RAPA + UCMSC-Exos group. Compared to the UCMSC-Exos group, early CD4^+^ T cell apoptosis in the RAPA group increased. Compared to the RAPA group, early CD4^+^ T cell apoptosis in the RAPA + UCMSC-Exos group decreased (Fig. 5C). Compared with the pSS group, early CD4^+^ T cell apoptosis in the UCMSC-Exos, HCQ, and HCQ + UCMSC-Exos groups decreased. There was no difference between the UCMSC-Exos and HCQ groups. Compared to the HCQ group, the early apoptosis in the HCQ + UCMSC-Exos group was further reduced (Fig. 5D). It is suggested that the autophagy inducer RAPA can induce early CD4^+^ T cell apoptosis. This was reversed by the addition of UCMSC-Exos to RAPA. In contrast, the autophagy inhibitor HCQ inhibited the early apoptosis, which was further reduced by the addition of UCMSC-Exos to HCQ. However, there was no difference in late apoptosis and overall apoptosis (Supplementary Fig S4). The results showed that UCMSC-Exos inhibited early CD4^+^ T cell apoptosis by inhibiting autophagy.

The ratios of Th1 and Th2 cells in the pSS, UCMSC-Exos, RAPA, and RAPA + UCMSC-Exos groups were similar and the Th1/Th2 in the RAPA group was lower than that in the pSS group. There was no difference in the proportion of Th1 and Th2 cells among the pSS, UCMSC-Exos, HCQ, and HCQ + UCMSC-Exos groups. The Th1/Th2 in the UCMSC-Exos, HCQ, and HCQ + UCMSC-Exos groups were lower than that in the pSS group. However, there was no significant difference in the Th1/Th2 ratio between the UCMSC-Exos and HCQ groups, and the HCQ and HCQ + UCMSC-Exos groups (Supplementary Fig S5).

Compared to the pSS group, the proportion of Th17 cells in the UCMSC-Exos and RAPA + UCMSC-Exos groups decreased, while that in the RAPA group showed a downward trend, but with no statistically significant difference. Furthermore, there was no difference between the UCMSC-Exos and RAPA groups, and the RAPA and RAPA + UCMSC-Exos groups (Fig. 5E). Therefore, to prove that UCMSC-Exos inhibited CD4^+^ T cell differentiation into Th17 cells by inhibiting autophagy, the effect of the autophagy inhibitor HCQ was further investigated. Compared to the pSS group, the proportion of Th17 cells in the UCMSC-Exos, HCQ, and HCQ + UCMSC-Exos groups decreased, with no difference of that between the UCMSC-Exos and HCQ groups. Compared to the HCQ group, the proportion of Th17 cells in the HCQ + UCMSC-Exos group was further decreased (Fig. 5G). This showed that HCQ can reduce the proportion of Th17 cells, and that the proportion was further reduced after adding UCMSC-Exos to HCQ, suggesting that UCMSC-Exos inhibits the differentiation of CD4^+^ T cells to Th17 cells by inhibiting autophagy.

Compared with the pSS group, the proportion of Tregs in the UCMSC-Exos group increased, while that in the RAPA group decreased; however, there was no difference in the RAPA + UCMSC-Exos group. Compared to the UCMSC-Exos group, the proportion of Tregs in the RAPA group decreased. Compared with the RAPA group, the proportion of Tregs in the RAPA + UCMSC-Exos group increased (Fig. 5F). Compared to the pSS group, the proportion of Tregs in the UCMSC-Exos, HCQ, and HCQ + UCMSC-Exos groups increased, with no difference of that between the UCMSC-Exos and HCQ groups. Compared with the HCQ group, the ratio of Tregs in the HCQ + UCMSC-Exos group was further increased (Fig. 5H). It is suggested that RAPA can reduce the proportion of Tregs. The addition of UCMSC-Exos to RAPA reversed the decrease in the proportion of Tregs. HCQ increased the proportion of Tregs, which was further increased by the addition of UCMSC-Exos to HCQ. This indicated that UCMSC-Exos promoted the differentiation of CD4^+^ T cells into Tregs by inhibiting autophagy.

Compared with the pSS group, the Th17/Treg in the UCMSC-Exos group decreased, that in the RAPA group showed an increasing trend but with no statistical difference, and that in the RAPA + UCMSC-Exos group decreased. Compared to the UCMSC-Exos group, the Th17/Treg increased in the RAPA group. Compared with the RAPA group, the Th17/Treg in the RAPA + UCMSC-Exos group was lower (Fig. 5E,F). Compared with the pSS group, the Th17/Treg in the UCMSC-Exos, HCQ, and HCQ + UCMSC-Exos groups decreased. There was no difference in the Th17/Treg between the UCMSC-Exos and HCQ groups. Compared to the HCQ group, the Th17/Treg in the HCQ + UCMSC-Exos group was further reduced (Fig. 5G,H). This suggests that the RAPA increased the Th17/Treg to a certain extent, and that Th17/Treg decreased after adding UCMSC-Exos to RAPA. The HCQ reduced the Th17/Treg, which was further reduced after adding UCMSC-Exos to HCQ. This indicated that UCMSC-Exos restored the Th17/Treg balance by inhibiting autophagy.

## Discussion

This retrospective study found that peripheral blood CD3^+^ T cells and CD4^+^ T cells decreased in pSS patients, suggesting that the decreased CD3^+^ T cells may be affected by the CD4^+^ T cells, which is consistent with the report by Mingueneau et al. (2016). This may be due to the migration of CD4^+^ T cells to the gland tissue (An et al. 2022), the presence of anti-CD4 antibody in the peripheral blood of pSS patients (Henriksson and Bredberg 2000), or the increased apoptosis of CD4^+^ T cells (Zeher and Szondy 1999). Meanwhile, this study found that CD4^+^ T cells were lower in patients with moderate and high disease activity, and negatively correlated with ESSDAI, with no correlation among other cell subsets. All the results indicated that CD4^+^ T cells play a key role in the pathogenesis of pSS.

This study subsequently showed that UCMSC-Exos inhibited the excessive proliferation of CD4^+^ T cells in pSS patients, blocked them in the G0/G1 phase, and prevented them from entering the S phase. Lee et al. reported that MSC-Exos can regulate the T cell cycle of the spleen in C57BL/6NHsd mice, resulting in G0/G1 phase cells increasing and S phase cells decreasing (Lee et al. 2020). Zeher et al. found that the apoptosis of CD4^+^ T cells in the peripheral blood of pSS patients increased and significantly correlated with disease activity and the degree of salivary gland lymphocyte infiltration, indicating that the abnormal function of peripheral blood T cells may reflect immunopathological events at the lesion site (Zeher and Szondy 1999). It has been shown that CD4^+^ T cell apoptosis also increases in the peripheral blood of patients with systemic lupus erythematosus (SLE) (Wen et al. 2013). The DNA complexes produced by apoptotic cells can induce the secretion of TNF-α and IFN-γ, and the nucleosomes in apoptotic cells can induce the production of autoantibodies (Wen et al. 2013). UCMSCs can inhibit excessive apoptosis of peripheral blood CD4^+^ T cells in SLE patients (Chen et al. 2016). Similar to these results, our study found that UCMSC-Exos inhibited excessive CD4^+^ T cell apoptosis in pSS patients.

This study found that UCMSC-Exos had no significant effect on the proportion of Th1 and Th2 cells. However, it reduced the proportion of Th17 cells, increased Treg proportion, and restored the Th1/Th2 and Th17/Treg balances. CD4^+^ T cells differentiate into different subsets and exert immune effects. Th1 cells secrete IFN-γ, TNF-α, and IL-2, activate macrophages, and inhibit the proliferation of Th2 cells. Th2 cells secrete IL-4 and IL-6, which activate B cells and inhibit Th1 cell proliferation. Th17 cells secrete IL-17A, IL-17F, and IL-22 to regulate Thl-mediated inflammation, while Tregs secrete TGF-β and IL-10 to induce immune tolerance (Verstappen et al. 2019). At present, the results of CD4^+^ T cell subsets in the peripheral blood of pSS patients differ. Sudzius et al. found no significant difference in the levels of Th1, Th2, and Th17 cells in pSS patients (Sudzius et al. 2013), while Kohriyama et al. found that the levels of Th1 cells decreased and Th2 cells were not significantly different (Kohriyama 2000). Another study reported that the levels of Th1 cells increased, Th2 cells decreased, while Th17 cells and Tregs were not significantly different (Loureiro-Amigo et al. 2021a, b). Yao et al. and Sudzius et al. showed that the Th17 cells increased and Tregs decreased (Yao et al. 2019; Sudzius et al. 2015). Dental pulp MSCs can reportedly reduce Th17 cells and increase Tregs in the peripheral blood of pSS patients, but they have no significant effect on Th1 and Th2 cells (Huang et al. 2017). UCMSCs also reduce the Th17 cells (Yao et al. 2019) and increase the Tregs (Liu et al. 2021; Yao et al. 2019). The results herein are consistent with the recently reported results that labial gland MSC-Exos reduce the proportion of Th17 cells and increase that of Tregs in pSS patients (Li et al. 2021).

The co-culture of labial gland MSC-Exos with the PBMC of pSS patients reportedly reduced the levels of IFN-γ, TNF-α, IL-6, and IL-17A and promoted the expression of IL-10 and TGF-β (Li et al. 2021). However, because these immune cells include CD8^+^ T cells, macrophages, and B cells, they can also secrete inflammatory factors, so it is difficult to fully reflect the role of MSC-Exos. Herein, CD4^+^ T cells from PBMC were isolated using immunomagnetic beads. We found that IFN-γ, TNF-α, IL-6, IL-17A, and IL-17F expressions were increased, while that of TGF-β was decreased in pSS patients, which was consistent with literature reports (Jin and Yu 2013). UCMSC-Exos reduced the IFN-γ, TNF-α, IL-6, IL-17A and IL-17F expressions, and promoted IL-10 and TGF-β expressions. IL-2 is secreted by Th1 cells, promotes the differentiation of Tregs, inhibits Th17 cells differentiation, and regulates the proliferation of T, NK, and B cells, which have both anti- and pro-inflammatory effects (Luo et al. 2018). The expression of IL-2 in the peripheral blood of pSS patients reportedly decreases (Luo et al. 2018). Short-term, low-dose IL-2 treatment achieved a certain effect (Miao et al. 2018). However, this study found that the expression of IL-2 increased in pSS patients with no difference following the UCMSC-Exos intervention. IL-4, secreted by Th2 cells, inhibits the differentiation of Th1 cells and promotes the proliferation and differentiation of B cells. There is reportedly no difference in the expression of IL-4 in the peripheral blood between pSS and nSS patients (van Woerkom et al. 2005), which was consistent with our study. IL-22 secreted by Th17 cells in the peripheral blood and salivary glands of pSS patients increased and was positively correlated with autoantibody (Loureiro-Amigo 2021b). However, our study found that there was no significant difference. Our results suggest that UCMSC-Exos can regulate the secretion of CD4^+^ T-related inflammatory factors, and have an immunomodulatory role.

Moreover, drugs for treating pSS based on experience rather than evidence-based medicine. So far, the HCQ is the most often used to treat pSS (Seror et al. 2021). HCQ exerts an immunomodulatory effect via interferes with Toll-like receptor (TLR) signaling and inhibits the type I IFN pathway (Sacre et al. 2012). HCQ was used as a controlled drug in many randomized controlled trials (Seror et al. 2021). In this study, HCQ and UCMSC-Exos were separately co-cultured with CD4^+^ T cells from the peripheral blood of pSS patients. The results found that both HCQ and UCMSC-Exos inhibited CD4^+^ T cell proliferation, early apoptosis, late apoptosis, overall apoptosis, decreasing the ratio of Th17, increasing Treg ratio and restoring the balance of Th17/Treg, as show in Fig. 5 and Fig S3. There was no significant difference between the HCQ and UCMSC-Exos group. The study indicated that UCMSC-Exos exerts an immunomodulatory effect on the CD4^+^ T cells.

Autophagy is an important mechanism that regulates the proliferation, apoptosis, and differentiation of CD4^+^ T cells (Clarke and Simon 2019). Herein, although the autophagy protein P62 was not detected, the results showed that the expressions of BECLIN1, LC3II/LC3I, *BECLIN1* mRNA, and *LC3II* mRNA were increased in peripheral blood CD4^+^ T cells of pSS patients, which suggested that the autophagy levels increased and that it can be inhibited by UCMSC-Exos. Alessandri et al. found that CD4^+^ T cell autophagy levels in the salivary glands of pSS patients were increased, and the level was positively correlated with disease activity and organ injury, but there was no difference in the peripheral blood (Alessandri et al. 2017; Colafrancesco et al. 2020). In rheumatoid arthritis, it was found that the CD4^+^ T cell autophagy level in the peripheral blood increased. Autophagy deficiency inhibits the proliferation of CD4^+^ T cells and promotes their apoptosis (van Loosdregt et al. 2016). The autophagy levels of peripheral blood T cells (Chen et al. 2016) are also increased in SLE. This inconsistency in literature may be related to whether or not the patients were treated with HCQ (Rainsford et al. 2015), which inhibits the fusion of autophagosomes and lysosomes, resulting in an inability to induce autophagy. Additionally, it may also be related to the heterogeneity of pSS patients, such as disease activity, course, and stages.

Moreover, this study showed that HCQ could inhibit the proliferation of CD4^+^ T cells, which was further reduced after the addition of UCMSC-Exos to HCQ, indicating that UCMSC-Exos inhibited the proliferation of CD4^+^ T cells by inhibiting autophagy. It has been found that after ATG3 (Jia and He 2011), ATG5 (Pua et al. 2007), and ATG7 (Jia et al. 2015) knockout in mice, the number of CD4^+^ T cells in the peripheral blood decreased. The specific mechanism may be that after CD4^+^ T cell autophagy inhibition, CDKN1B aggregation of a negative regulator of cyclin-dependent kinase is promoted, which causes CD4^+^ T cells to not enter the S phase after T cell receptor stimulation (Jia et al. 2015). Interestingly, we found that CD4^+^ T cell proliferation was inhibited after RAPA-induced autophagy, contrary to the expected results of enhanced proliferation with increased autophagy levels. This may be related to the characteristics of RAPA, which inhibits T cell proliferation by affecting glycolysis and lipid and protein synthesis (Huang H, 2020).

Herein, RAPA induced the early apoptosis of CD4^+^ T cells in pSS patients, and the addition of UCMSC-Exos reversed the RAPA-induced early apoptosis. Similarly, HCQ inhibited the early apoptosis of CD4^+^ T cells, which was further reduced after UCMSC-Exos was added to HCQ. These results indicate that UCMSC-Exos inhibited the early apoptosis of CD4^+^ T cells by inhibiting autophagy. Chen et al. reported that the autophagy of peripheral blood CD4^+^ T cells in SLE patients increased, and that RAPA increased CD4^+^ T cell apoptosis, whereas the autophagy inhibitor 3-methyladenine reduced apoptosis (Chen et al. 2016). Our study are similar to these results. However, Chen et al. found that the autophagy inhibitor chloroquine (CQ) promoted the apoptosis of CD4^+^ T cells (Chen et al. 2016). The results of our study differed. This is because of the complex relationship between autophagy and apoptosis. Autophagy stimulated by damaged digestive organelles or misfolded proteins generates new materials to provide cells with energy and nutrition to prevent apoptosis (Ploumi et al. 2022). However, autophagy increases under nutrient deficiency to maintain the intracellular ATP levels, whereas exposing intracellular phosphatidylserine to the extracellular release of apoptotic signals instead promotes apoptosis. The specific mechanism involved in the complex relationship between autophagy and apoptosis may be further elucidated.

Autophagy deficiency has been shown to result from *Beclin1* knockout in CD4^+^ T cells, which inhibits Th1 and Th2 cells differentiation (Kovacs et al. 2012). Another study found that *Atg5* knockout did not affect Th1 or Th2 cell differentiation (Rivera Vargas et al. 2017). Herein, UCMSC-Exos did not regulate the Th1/Th2 imbalance via the autophagy pathway, which may be regulated through other pathways. After the HCQ intervention, Th17 cells decreased, Tregs increased, and Th17/Treg decreased. The above indicators were improved after UCMSC-Exos were added to HCQ, indicating that UCMSC-Exos regulates the differentiation of CD4^+^ T cells into Th17 and Tregs and restores the Th17/Treg balance through autophagy. Interestingly, the proportion of Th17 and Tregs decreased after RAPA intervention, while Th17/Treg cells showed an increasing trend. After the addition of UCMSC-Exos to RAPA, the proportion of Th17 cells did not change significantly; however, the proportion of Tregs increased and the Th17/Treg ratio decreased. This also suggests that UCMSC-Exos may regulate the Th17/Treg imbalance via autophagy. RAPA induces autophagy by inhibiting mTOR, and mTOR enhances Th17 cell differentiation and inhibits Treg differentiation (Huang et al. 2020). RAPA can reduce the proportion of Th17 cells in the peripheral blood of SS patients, increase the proportion of Tregs, and reduce the Th17/Treg ratio (Zhang et al. 2021). When RAPA-pretreated MSCs were co-cultured with CD4^+^ T cells, Tregs were induced (Cen et al. 2019). These results are inconsistent with those previously reported in literature. Hence, to further confirm our results, the sample size can be further expanded, or different methods can be used.

## Conclusions

This study demonstrated that peripheral blood CD4^+^ T cells decreased in pSS patients, especially in patients with moderate and high disease activity, and negatively correlated with ESSDAI. In vitro studies showed that UCMSC-Exos can regulate the abnormal proliferation, apoptosis, and differentiation of CD4^+^ T cells in the peripheral blood of pSS patients, and this is achieved by regulating the autophagy pathway in CD4^+^ T cells. However, only the immunomodulatory effect of UCMSC-Exos on CD4^+^ T cells in peripheral blood was investigated. Exocrine glands are the target organs of pSS. Owing to the small volume of labial glands obtained from patients, it was difficult to select CD4^+^ T cells for the experiment. Therefore, the effects of UCMSC-Exos will be further studied in NOD mice. Additionally, the relationship between autophagy and apoptosis of CD4^+^ T cells, how UCMSC-Exos regulates CD4^+^ T cell autophagy, and the contents that play a role in this process need to be further studied and clarified.

## Supplementary Information

Below is the link to the electronic supplementary material.

Supplementary file1 (DOCX 5044 kb)

## Data Availability

The dataset supporting the conclusions of this manuscript is included within the manuscript.

## Abbreviations

APC: Allophycocyanin
CFA: Complete Freund’s adjuvant
CIA: Collagen-induced arthritis
CII: Type II collagen
EULAR: European League Against Rheumatism
ESSDAI: EULAR SS disease activity index
EVs: Extracellular vesicles
Exos: Exosomes
FITC: Fluorescein isothiocyanate
FOXP3: Forkhead box P3
GADPH: Glyceraldehyde-3-phosphate dehydrogenase
HE: Hematoxylin and eosin
HSP70: Heat shock protein 70
hUCMSCs: Human umbilical cord mesenchymal stem cells
MPs: Microparticles
MTX: Methotrexate
IL: Interleukin
OD: Optical density
PBMCs: Peripheral blood mononuclear cells
PI: Propidium iodide
RA: Rheumatoid arthritis
RORγt: Etinoic acid receptor-related orphan receptor gamma tRT-PCR: reverse transcription-polymerase chain reaction
TGF-β: Transforming growth factor-β
Th17: T helper cell 17
Treg: Regulator T cell
