# Supplementary material for: Immunomodulatory effects of umbilical mesenchymal stem cell-derived exosomes on CD4+ T cells in patients with primary Sjögren's syndrome
Source: Inflammopharmacology. 2023 Apr 3;31(4):1823–38. doi: 10.1007/s10787-023-01189-x (PMC10352432; doi:10.1007/s10787-023-01189-x)
Supplement: Supplementary file 1 — Supplementary file1 (DOCX 5044 kb) [file 10787_2023_1189_MOESM1_ESM.docx]

**Supplementary**

**1. Materials and Methods**

1.1 Population

Table. S1 Characteristics of pSS patients and HC

| **Characteristics** | pSS* | HC* |
| --- | --- | --- |
| age, mean±SD*, years | 49.51 ±10.97 | 44.83±6.09 |
| Gender (Female/Male) | 68/4 | 22/2 |
| duration, M (Q25, Q75)*, years | 2.00 (1.00, 6.00) | - |
| ESSDAI (5–13)*, n (%) | 57 (79.2%) | - |
| ESSDAI (≥14), n (%) | 15 (20.8%) | - |

*ESSDAI, European League Against Rheumatism Sjögren’s syndrome disease activity index; HC, healthy control; M, median; pSS, primary Sjogren's Syndrome; Q, quartile; SD, standard deviation

1.2 Isolation and identification of UCMSCs and UCMSC-Exos

Table S2 Preparation of the SDS-PAGE gel

| reagent | 10% separation gel  （10ml） | 5% spacer gel  （4ml） |
| --- | --- | --- |
| Three times distilled water | 4.00 | 2.70 |
| 30% acrylamide | 3.30 | 0.67 |
| 1.5M Tris-HCl（PH8.8） | 2.50 | - |
| 1.0M Tris-HCl（PH6.8） | - | 0.50 |
| 10%SDS | 0.10 | 0.04 |
| 10%APS | 0.10 | 0.04 |
| TEMED | 0.004 | 0.004 |

1.3 Cell Counting Kit-8 (CCK8) method

Immunomagnetic beads sorted peripheral blood CD4^+^ T cells from six healthy control (HC) and six pSS patients. The 96-well plate were coated with anti-CD3 antibody (5 μg/mL; BioLegend, CA, USA) and incubated at 37 ℃ for 2 to 4 h. Then, the anti-CD3 antibody was aspirated. The 96-well plate were added CD4^+^ T cell suspension resuspended in RPMI 1640 medium (Gibco, Carlsbad, CA, USA), adjusted cells to 1×10^5^/mL, added PBS or UCMSC-Exos resuspended in PBS, and adjusted the total solution to 100 μL, namely HC (PBS intervention), pSS (PBS intervention), UCMSC-Exos at different concentrations group (30 μg/mL, 60 μg/mL, and 90 μg/mL, respectively). The CD4^+^ T cells in the pSS and UCMSC-Exos groups were obtained from the same patient. The anti-CD28 antibody (2 μg/mL; BioLegend) was added and incubated for 72 h. Then, CCK8 solution (10 μL; Solarbio, Beijing, China) was added and incubated for 4 h. The absorbance of each well (OD at 450 nm) was measured by enzyme-labeled instrument (Agilent Technologies, Winooski, VT, USA).

1.4 Detection of autophagy levels

Table S3 Preparation of the SDS-PAGE gel

| reagent | 15% separation gel（10ml） | 5% spacer gel  （4ml） |
| --- | --- | --- |
| Three times distilled water | 2.30 | 2.70 |
| 30% acrylamide | 5.00 | 0.67 |
| 1.5M Tris-HCl（PH8.8） | 2.50 | - |
| 1.0M Tris-HCl（PH6.8） | - | 0.50 |
| 10%SDS | 0.10 | 0.04 |
| 10% ammonium peroxydisulfate（APS） | 0.10 | 0.04 |
| TEMED | 0.004 | 0.004 |

Table S4 Primer sequences for the autophagy genes

| gene | primers（5’-3’） | bp | Tm |
| --- | --- | --- | --- |
| Beclin 1 | F: ATG CAG GTG AGC TTC GTG TG | 20 | 58.4 |
|  | R: CTG GGC TGT GGT AAG TAA TGG A | 22 | 56.7 |
| LC3Ⅱ | F: AGT TGG CAC AAA CGC AGG GTA | 21 | 59.9 |
|  | R: TTA GGA GTC AGG GAC CTT CAG CA | 23 | 59.8 |
| β-actin | F: TGG CAC CCA GCA CAA TGA A | 19 | 57.8 |
|  | R: CTA AGT CAT AGT CCG CCT AGA AGC A | 25 | 57.9 |

**2.** **Results**

2.1 The purity of CD4^+^ T cells


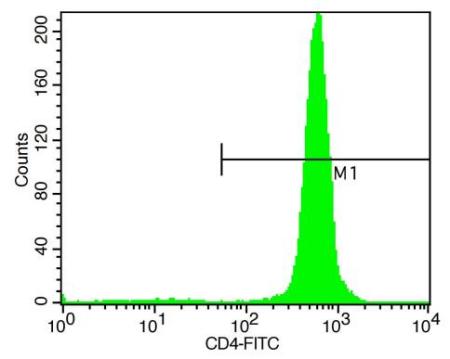


Fig S1. The purity of CD4^+^ T cell sorted by immunomagnetic beads was 98.29%.

2.2 CD4^+^ T cell proliferation was determined by the CCK8 method

Compared to the HC group, the proliferation of peripheral blood CD4^+^ T cells in pSS patients was increased. There was no significant difference between the pSS and UCMSC-Exos (30 μg/mL) groups. The proliferation CD4^+^ T cells in UCMSC-Exos (60 μg/mL) and UCMSC-Exos (90 μg/mL) intervention was decreased, and the 90 μg/mL was better than 60 μg/mL. Moreover, there was no significant difference between the UCMSC-Exos (90 μg/mL) and the HC groups.





## Fig S2. UCMSC-Exos regulated the proliferation of CD4^+^ T cells in pSS patients. **P*<0.05, ****P*<0.001, NS *P*>0.05.


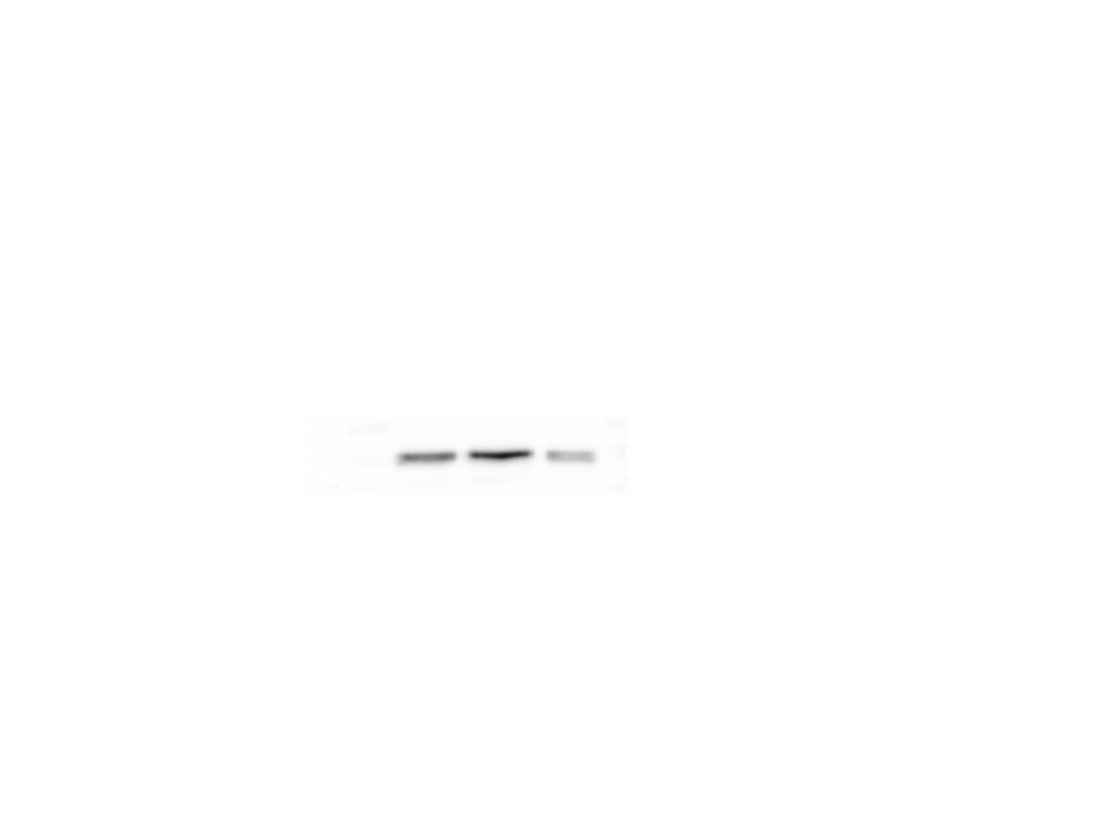


**pSS**

**RAPA**

**HCQ**

**Beclin1**

**LC3Ⅰ**

**LC3Ⅱ**

**β-actin**

**52KD**

**14KD**

**42KD**

**16KD**


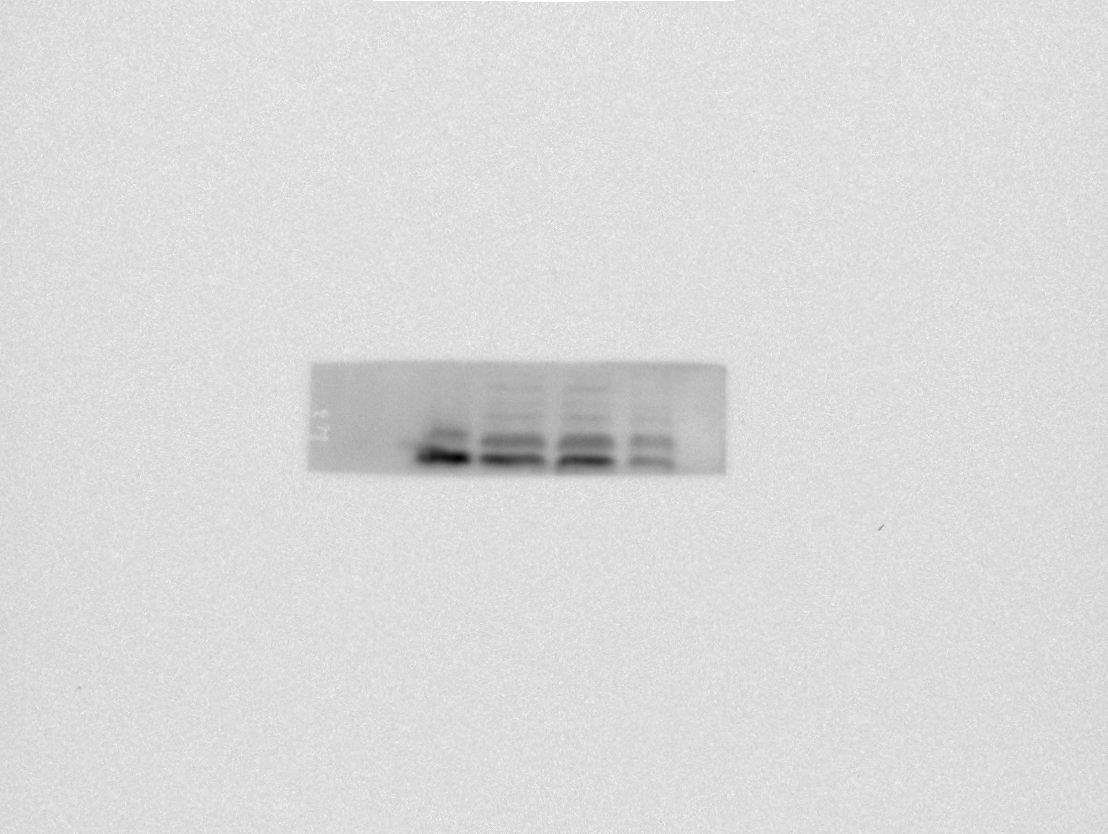


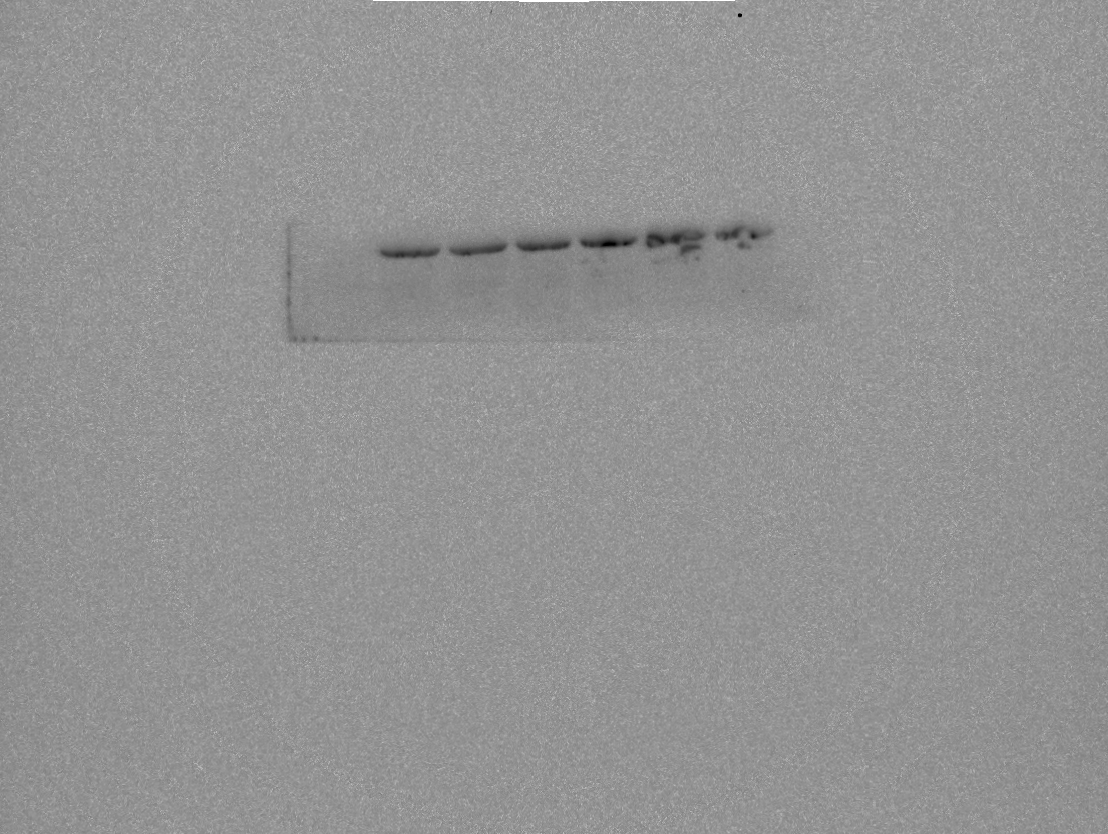


Fig S3. Effects of RAPA and HCQ on CD4^+^ T cells autophagy levels. RAPA increased the levels of Beclin1 and LC3II/LC3I on peripheral blood CD4^+^ T cells of pSS patients, while HCQ decreased the autophagy levels.











A

B

Fig S4. UCMSC-Exos regulated the apoptosis of CD4^+^ T cells in pSS patients by inhibiting the autophagy. **A.** Effects of UCMSC-Exos on CD4^+^ T cells apoptosis after autophagy induction by RAPA. **B.** Effects of UCMSC-Exos on CD4^+^ T cells apoptosis after autophagy inhibited by HCQ. **P*<0.05, ***P*<0.01, ****P*<0.001.

A

B















Fig S5. UCMSC-Exos regulated the differentiation of CD4^+^ T cells in pSS patients by inhibiting the autophagy. **A.** Effects of UCMSC-Exos on CD4^+^ T cells differentiation after autophagy induction by RAPA. **B.** Effects of UCMSC-Exos on CD4^+^ T cells differentiation after autophagy inhibited by HCQ. **P*<0.05, ***P*<0.01.
